# RETRACTION: Utility of Dexmedetomidine on Surgical Site Wound Pain Undergoing Thoracoscopic Surgery: A Meta‐Analysis

**DOI:** 10.1111/iwj.70443

**Published:** 2025-03-26

## Abstract

**RETRACTION:** LiM. , ZhangK. , LuH. , LiangY. , ZhangY. , and FengG. , “Utility of Dexmedetomidine on Surgical Site Wound Pain Undergoing Thoracoscopic Surgery: A Meta‐Analysis,” International Wound Journal 21, no. 4 (2024): e14629, 10.1111/iwj.14629.38156707 PMC10961883

The above article, published online on 29 December 2023, in Wiley Online Library (http://onlinelibrary.wiley.com/), has been retracted by agreement between the journal Editor in Chief, Professor Keith Harding; and John Wiley & Sons Ltd. Following an investigation by the publisher, all parties have concluded that this article was accepted solely on the basis of a compromised peer review process. The editors have therefore decided to retract the article. The authors did not indicate their agreement with the retraction.

**RETRACTION:** M. Li , K. Zhang , H. Lu , Y. Liang , Y. Zhang , and G. Feng , “Utility of Dexmedetomidine on Surgical Site Wound Pain Undergoing Thoracoscopic Surgery: A Meta‐Analysis,” International Wound Journal 21, no. 4 (2024): e14629, 10.1111/iwj.14629.38156707 PMC10961883

The above article, published online on 29 December 2023, in Wiley Online Library (http://onlinelibrary.wiley.com/), has been retracted by agreement between the journal Editor in Chief, Professor Keith Harding; and John Wiley & Sons Ltd. Following an investigation by the publisher, all parties have concluded that this article was accepted solely on the basis of a compromised peer review process. The editors have therefore decided to retract the article. The authors did not indicate their agreement with the retraction.

